# Risk assessment of waterborne infections in Enugu State, Nigeria: Implications of household water choices, knowledge, and practices

**DOI:** 10.3934/publichealth.2020050

**Published:** 2020-08-06

**Authors:** Onyekachi Juliet Okpasuo, Ifeanyi Oscar Aguzie, Anunobi Toochukwu Joy, Fabian C Okafor

**Affiliations:** 1 Parasitology and Public Health Research Laboratory, University of Nigeria, Nsukka, Nigeria; 2 Science Laboratory Technology Department, Federal Polytechnic, Idah, Kogi State, Nigeria; 3 Ecology and Environmental Biology, University of Nigeria, Nsukka, Nigeria

**Keywords:** water quality, safe drinking water, poor hygiene, waterborne diseases

## Abstract

This research investigated the prevalence of waterborne infections (WBIs) and the risks associated with household drinking water choices, knowledge, and practices. A cross-sectional multi-stage sampling research design was employed. A well-structured questionnaire was used to sample 403 individuals representing 115 household; and stool samples collected and subjected to standard parasitic and bacterial diagnostic methods. From the 403 samples, 344 (85.4%) were positive for at least one waterborne pathogen of nine isolates: *E. coli* (38.0%), *Giardia* *lamblia* (35.2%), *E. histolytica* (33.0%), *Salmonella* *typhi* (19.9%), *Proteus* spp. (13.2%), *Shigella* *dysentery* (9.4%), *Klebsiella* spp. (7.4%), *Enterobacter* spp. (5.5%), and *Cryptosporidium* spp. (5.2%). Prevalence of WBIs was >75% in all age groups, but decreased with age. Prevalence of WBIs was >80% in all communities. Risk was not biased by gender. Odds of infection from public well (OR = 2.487; CI_95_: 1.296–4.774) and borehole/vendor (OR = 2.175; CI_95_: 1.231–3.843) users was over two times greater than non-users. Risk of WBDs was significantly reduced by 60% in sachet water drinkers (OR = 0.392; CI_95_: 0.217–0.709; p < 0.05). Surprisingly, river/stream water users had a significant reduced risk of WBDs than non-users (OR = 0.335; CI_95_: 0.150–0.749; p < 0.05). Poor hygiene was the most important determinant of WBIs; poor sanitary practice increased odds of WBIs by 400% (OR = 4.945; CI_95_: 2.358–10.371; p < 0.05). This study shows that most household water choices are vulnerable to contamination at many points in their journey from source to mouth; and advocates adequate provision of safe water, “point of use” household water treatment, and good storage methods to effectively curb WBIs.

## 1. Introduction

Water is a key determinant of sustainable development that should be carefully managed to ensure sustainable human health. In homes, water is indispensably required for domestic needs such as drinking, bathing, and cooking [1]. Failure to ensure the safety of water may expose the community to the risk of disease outbreaks. Thus, an assured supply of water both qualitatively and quantitatively for these purposes will greatly improve health, economics, and social wellbeing. The World Health Organization [2] reports that over 2.6 billion people lack access to clean water, which is responsible for about 2.2 million deaths annually, of which 1.4 million are children. Nigeria is facing many waterborne related challenges and it is disheartening that majority of the population does not have access to good drinking water and must rely on the use of unsafe sources to satisfy basic needs [3]. Most households, in a bid to survive, have resorted to utilizing any available water within their community regardless of its quality. This is coupled with the widespread lack of appropriate knowledge of how to treat and adequately preserve water to prevent further contamination [3]. Accessible water sources in Nigeria include boreholes, wells, springs, streams, lakes, rainwater, and government distributed municipal water. Most of which fall within the category of unimproved drinking water sources as characterized by WHO/UNICEF [4].

Waterborne diseases (WBDs) are those diseases which generally arise from contamination of water by human or animal feces and/or urine infected by pathogenic viruses, bacteria, protozoa, or helminths and are directly transmitted when unsafe water is drunk [5]. Worldwide, it has been shown that WBDs are responsible for over 2–3 million deaths yearly [6]. WBDs are very rampant, especially in sub-Saharan Africa, due to lack of access to clean water and poor sanitation. WBD outbreaks have occurred either when drinking water supplies were not adequately treated after contamination with surface water or when surface waters contaminated with enteric pathogens have been used for recreational purpose [7]. Many rivers, streams and wells worldwide are affected by fecal contamination leading to increased health risks to persons exposed to the water and degradation of recreational and drinking water quality [8]. Recently, cholera and other diarrheal disease outbreaks were reported from the developing countries, Cameroon, Nigeria, Tanzania, Zimbabwe, Somalia, and South Africa. These outbreaks were attributed to contaminated drinking water and inadequate sanitation [2],[9]–[13]. In a study on the prevalence of waterborne pathogens, Fletcher et al. [14] reported that *Campylobacter* spp., *E. coli* pathotypes and *Shigella* spp. are frequently detected in children from developing regions while adults are predominantly affected by *Cryptosporidium* sp., *Salmonella* and pathogenic *E. coli*.

Several factors could affect the transmission of WBD pathogens through household drinking water sources. They include environmental factors such as changing climate, available water supply, and sanitation facilities. A lack of safe water supply, inadequate excreta disposal facilities, poor hygiene, poor living conditions, and flooding after heavy rainfall promote the distribution and contamination of drinking water sources [15]. These factors correspond with the retrospective analysis of WBD in Enugu State which showed a high distribution of WBD in the area [16]. Additionally, the population density of both the household and community is another factor that has contributed to the contamination of drinking water sources [17]. Nigeria has recorded an increased population rate of 3.0%, this high rate will affect family size and indirectly affect household utility factor in relation to water demand [17]. This implies that a household demand for drinking water would increase as family size increases, hence most households who cannot afford the cost of quality drinking water because of family size increase will resort to questionable water sources. Finally, the individual's knowledge, attitude, and practices towards drinking water choices, water treatment, and storage are important factors that determine the risk of contamination of drinking water.

Household drinking water can be obtained from heterogeneous sources which are dependent on several factors such as price, availability, quality, distance to water sources, perceived risk, and knowledge of water treatment methods. There is very little documentation about the link between household drinking water choices, knowledge, attitudes, and practices (KAP) to waterborne diseases in South-eastern, Nigeria. This situation perpetrates inadequate awareness of the link and associated risks between the burden of WBD pathogens and household drinking water choices and KAP. Therefore, this study was designed to investigate the prevalence and associated risk of waterborne diseases in Enugu Urban, Enugu State, Nigeria. These findings will be beneficial as it will provide baseline information on household drinking water choices, knowledge, and practices. It will also evaluate the importance of adequate supply of safe water to households, as well as provide information on human health risks deriving from household dependence on unprotected water bodies. This work also has the potential to help in the formulation of general water use, storage, and treatment policies, especially with regards to WBDs in Nigeria.

## 2. Materials and methods

### 2.1. Study area

Enugu State is one of the major states in South-eastern Nigeria. It is located within latitude 60.00′N and 70.00′N and longitudes 70.00′E and 70.45′E [18]. Enugu urban has three Local Government Areas (LGAs), namely, Enugu East, Enugu North, and Enugu South. These LGAs have the communities, Abakpa, Trans-Ekulu, Nike, GRA, Ogui, Asata, New Heaven, Obiagu, Ogbete, Iva Valley, Independence Layout, Achara Layout, Ugwuaji, Maryland, Awkanaw, Uwani, Agbani, and Coal Camp. These communities are characterized as urban, peri-urban, sub-urban and urban slums. Enugu Urban falls within the humid tropical rainforest belt of South-eastern Nigeria. The rivers and streams which flow from the Udi hills dissect the study area into several sections. Thus, there are rivers such as Ekulu, Idaw, Asata and Nyaba Rivers which separates Enugu South from Nkanu East, these rivers have many tributaries.

The population density per square kilometer in Enugu Urban is approximately 1741 person/km^2^ [19]. Pipe-borne water supply are infrequent in most areas of the city. The State Water Corporation employs what it termed the administrative method approach in the allocation of water supply to consumers; this is the estimation of water demand based on past consumption data, i.e. based on established records of water consumption of each area or sector [20]. Despite the use of this method, many sectors are faced with an inconsistent or complete lack of supply making the inhabitants resort to other sources of water such as wells, streams, rivers and some unhygienic water storage methods. Sanitation in some parts of Enugu urban is commendable but very poor in some peri-urban slums and sub-urban areas which includes Abakpa, Emene, Obiagu, Ogbete, Asata, Uwani, and Coal camp.

### 2.2. Ethical clearance and advocacy visit

The study commenced after obtaining a formal ethical clearance from the University of Nigeria Teaching Hospital. The ethical approval number is NHREC/05/01/2008B-FWA00002458-1RB00002323. This was followed by an advocacy visit to sensitize the households and seek their consent to participate in the research.

### 2.3. Research design and data collection

A cross-sectional research design involving a multi-stage sampling method was adopted for data collection. The selected communities were Abakpa, Trans-Ekulu, Ogui, Asata, Awkunanaw and Uwani, representing the three LGA in Enugu Urban. The houses in the communities were selected by systematic random sampling. The data used was obtained from analysis of stool samples, a well-structured questionnaire, and complementary interview. A total of 403 stool samples were collected with a clearly labelled standard collection tube. The questionnaire captured respondents' demographics, drinking water choices, motivations for such choices, and knowledge and practices related to drinking water hygiene and choices. Sample collection lasted from January to August 2017.

### 2.4. Examination of stool samples and identification of pathogens

The freshly collected stool samples were immediately taken to the Parasitology and Public Health Laboratory, University of Nigeria, Nsukka for examination. The stool samples were analyzed using formol-ether concentration techniques and culture methods for parasite and bacterial infection diagnosis, respectively. The pathogens were identified with standard identification keys highlighted by Vandepitte et al. [21] and Cheesbrough [22].

### 2.5. Statistical analysis

Data collected was collated and entered into Microsoft Excel 2010 and exported to SPSS version 21 for statistical analysis. Chi-square analysis was used to determine the prevalence of waterborne pathogens in relation to demographic characteristics, age, sex, community and LGAs. A summary of frequency of respondents for different demographic and water-choice related characteristics were either provided as cumulative (i.e. adding up to 100%) where necessary, or simply as the percentage of participants that answered in the affirmative for categories of interests (and does not necessarily add up to 100%). The risks of WBDs presented in a crude odd ratio were estimated using binary logistic regression with the variable dummy coded as 0 for negative and 1 for positive responses. The negative response was used as the reference category and odds for positive responses presented. Level of significance was set at p < 0.05.

## 3. Results

### 3.1. Overall prevalence of waterborne diseases in Enugu Urban

Out of 403 individuals whose stool samples were examined for presence of waterborne pathogens, 344 (85.4%) were positive for at least one waterborne microbial agent. The prevalence according to age, sex, community and LGA are shown in Table 1. The prevalence of waterborne infections (WBIs) was above 75% across all age groups but lower in older compared to younger age groups. The disparity in prevalence was not significant statistically (p > 0.05). Prevalence of WBIs was above 80% in all communities sampled (Table 1); highest in Enugu North (89.0%) and least in Enugu South (83.1%); and high in males and females (86.7% vs. 84.2%).

**Table 1.** Overall prevalence of waterborne diseases in Enugu Urban, Nigeria.

Demographic variables	Number examined	Number infected	Prevalence (%)
Age (years)			
0–5	96	87	90.6
6–15	93	80	86.0
16–25	56	48	85.7
26–35	66	56	84.8
36–45	48	39	81.2
≥46	44	34	77.3
			χ^2^ = 5.138, p = 0.400
Sex			
Male	184	154	83.7
Female	219	190	86.8
			χ^2^ = 0.750, p = 0.389
Communities			
Abakpa	75	65	86.7
Trans-Ekulu	59	48	81.4
Ogui	59	54	91.5
Asata	68	59	86.8
Awkunanaw	72	59	81.9
Uwani	70	59	84.3
			χ^2^ = 3.498, p = 0.624
Local Governments			
Enugu East	134	113	84.3
Enugu North	127	113	89.0
Enugu South	142	118	83.1
			χ^2^ = 2.024, P = 0.363

### 3.2. Etiological agents of WBD in Enugu Urban

**Figure 1.**
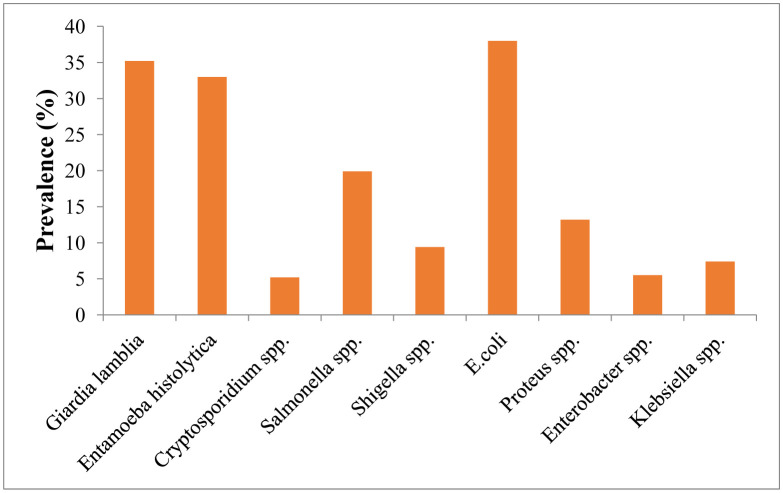
Overall prevalence of waterborne etiological agents in Enugu Urban, Nigeria.

The study identified WBD etiological agents belonging to nine different genera namely, *Giardia, Entamoeba, Cryptosporidium*, *Salmonella, Shigella, Escherichia, Proteus, Enterobacter* and *Klebsiella* with prevalence ranging from 5.2 to 38.0% (Figure 1). The most prevalent was *E. coli*, while the least was *Cryptosporidium* *parvum*.

Out of the 403 individuals examined, 265 (65.8%) were co-infected with two or three of the waterborne microbes. Overall, 218 (54.1%) had co-infections of two different pathogenic agents while 47(11.7%) had co-infections of three. Prevalence of each one of the observed co-infections was below 7.0%. *G. lamblia/E. histolytic* co-infection of 6.7% was the highest (Supplementary Table S1). The intensity of infection based on the scanty (+), moderate (++), and severe (+++) categorization is presented as supplementary Figure S1.

### 3.3. Seasonal prevalence of waterborne etiological agents in Enugu Urban

**Figure 2.**
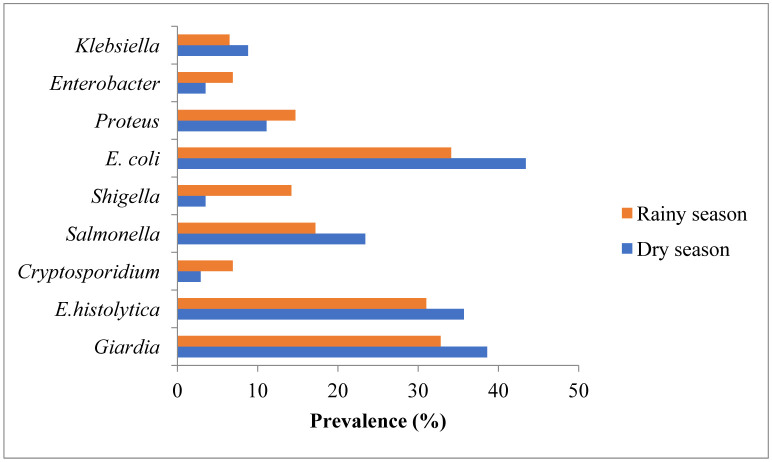
Seasonal prevalence of waterborne etiological agents in Enugu urban, Nigeria.

A total of 171 individuals were examined during the dry season, out of which 149 (87.1%) tested positive while out of 232 individuals sampled during the rainy season, 195 (84.1%) tested positive. Difference in prevalence between the seasons was small overall (p > 0.05; Figure 2).

### 3.4. Demographic characteristics of survey respondents

All of the 403 study participants belonging to 115 households responded to the questionnaire and interview. The overall demographic characteristics of the respondents are shown in Table 2. Most of the respondents were between 16 and 25 years old (60.8%). There were 10% more female respondents than male (54.3% vs. 45.7%). Females and males were approximately equally represented at community and LGA levels.

**Table 2.** Overall demographic characteristics of study participants, Enugu Urban, Nigeria.

Variables		Frequency (%)
Sex	Male	184(45.7)
	Female	219(54.3)
Age (year)	0–5	96(23.8)
	6–15	93(23.1)
	16–25	56(13.9)
	26–35	66(16.4)
	36–45	48(11.9)
	45≧	44(10.9)
Community	Abakpa	75(18.6)
	Trans-Ekulu	59 (14.6)
	Ogui	59(14.6)
	Asata	68(16.9)
	Uwani	72(17.9)
	Awkunanaw	70(17.4)
LGA	Enugu East	134(33.3)
	Enugu North	127(31.5)
	Enugu South	142(35.2)

### 3.5. Respondents' water choices, knowledge, and practices

**Table 3.** Respondents' water choices of sampled households in Enugu Urban, Nigeria.

Water Choices	Categories	Frequency (%)
Drinking water choice	Municipal	101(25.1)
	Public well	155(38.5)
	Private well	16(4.0)
	Borehole/vendors	216(53.6)
	Stream/rivers	32(7.9)
	Rainwater	74(18.4)
	Sachet/bottle water	87(21.6)
Reason for the choice	Price	74(18.4)
	Distance	5(1.2)
	Quality and reliable	63(15.6)
	Available	365(90.6)
	Average perception of water safety	108(26.8)
Knowledge		
Knowledge of Waterborne diseases	Diarrhea	380(94.3)
	Dysentery	110(27.3)
	Cholera	118(29.3)
	Typhoid	201(49.9)
	Others	47(11.7)
	Do not know	15(3.7)
Knowledge of factors causing the diseases	Dirty environments	269(66.7)
	Unhygienic practices	221(54.8)
	Drinking contaminated water	388(96.3)
	No idea	15(3.7)
Knowledge of water treatment	Have knowledge of water treatment methods	108(26.8)
	Have no knowledge of water treatment methods	256(63.5)
Practices		
Water storage practices	Plastic bucket with lid	134(33.3)
	Plastic bucket without lid	78(19.4)
	Tanks	120(29.8)
	Pots	14(3.5)
	Gallons	36(9.4)
	None (sachet water users)	19(4.7)
Water treatment practices	Boiling	112(27.8)
	Sedimentation	31(7.7)
	Filtration	32(7.9)
	Chlorination	6(1.5)
	Other methods	6(1.5)
Personal hygiene	Very good	52(12.9)
	Good	169(41.9)
	Poor	171(42.4)
	Very poor	11(2.7)

The household water choices are presented in Table 3. Borehole water which is supplied by vendors (borehole/vendors) was the major drinking water choice (53.6%). This was followed by public well (38.5%) and Municipal (25.1%). Private well was the least adopted (4.0%). Nearly all respondents (90.6%) indicated that the reason for their water choice was availability. Quality/reliability and price was only considered relevant by 15.6% and 18.4% respectively. Safety of the water was also given a low consideration (26.8%).

Most Enugu urban inhabitants had some knowledge of the four WBDs. Only 3.7% of the respondents acknowledged being aware of WBDs. However, knowledge about the four WBDs widely differed. The majority considered diarrhea (94.3%) a WBD; which was followed from a distance by typhoid fever (49.9%). Less than 30% of the respondents considered dysentery and cholera a WBD.

Knowledge of water treatment practices was low among the people; 63.5% had no knowledge about water treatment methods compared to 26.8% who had. Plastic bucket with lid (33.3%) and tanks (29.8%) were the major water storage mediums used by households. Boiling of water was the main treatment practices employed (27.8%). More than half of respondents have good personal hygiene.

### 3.6. Waterborne disease risks associated with water choices, knowledge and practices

The odds of infection with one or more of the waterborne microbes isolated from the study participants are presented in Table 4. The odds of infection from a public well (OR = 2.487; CI_95_: 1.296–4.774) or borehole/vendor (OR = 2.175; CI_95_: 1.231–3.843) were over two times more compared to non-users (p < 0.05). The risk of WBDs was significantly reduced by approximately 70% in sachet water drinkers compared to the non-drinkers (OR = 0.392; CI_95_: 0.217–0.709). Surprisingly, river/stream water users had a significantly reduced risk (only 68.8%) of WBDs compared to the non-users; (OR = 0.335; CI_95_: 0.150–0.749; p < 0.05). Municipal, private well and rainwater users had similar odds of WBDs compared to their respective non-users.

**Table 4.** Risk of waterborne diseases relative to water choices, knowledge, and practices in Enugu Urban, Nigeria (number of respondents = 403).

Water choices	No infected (%)	Odd ratio	95% CI
Drinking water choices			
Municipal	85(84.2)	0.882	0.473–1.646
Private well	13(81.2)	0.733	0.202–2.655
Public well	142(91.6)	2.487	1.296–4.774*
Borehole/vendors	194(89.8)	2.175	1.231–3.843*
Stream/rivers	22(68.8)	0.335	0.150–0.749*
Rainwater	66(89.2)	1.513	0.685–3.342
Sachet water	65(74.7)	0.392	0.217–0.709*
Reason for the choice			
Price	62(83.8)	0.861	0.431–1.718
Distance	5(100)	0.852	0.818–0.857
Quality and reliable	48(76.2)	0.476	0.246–0.921*
Available	314(86)	1.642	0.713–2.781
Average perception of water safety	89(82.4)	0.745	0.404–1.335
Knowledge			
Knowledge of waterborne diseases			
Diarrhea	321(84.5)	1.184	1.134–1.236*
Dysentery	90(81.8)	0.691	0.381–1.247
Cholera	98(83.1)	0.777	0.432–1.398
Typhoid	171(85.1)	0.955	0.550–1.660
Others	41(87.2)	1.195	0.484–2.955
Do not know	15(100)	0.848	0.813–0.884
Knowledge of factors causing the disease			
Dirty environments	186(82.2)	0.807	0.461–1.415
Unhygienic practices	329(84.8)	1.179	1.131–1.230
Drinking contaminated water	15(100)	0.848	0.813–0.848
No idea			
Knowledge of water treatment			
Have Knowledge of water treatment methods	114(77.6)	0.391	0.223–0.684
Practices			
Water storage practices			
Plastic bucket with lid	114(85.1)	0.967	0.539–1.733
Plastic bucket without lid	71(91.0)	1.932	0.841–4.436
Tanks	105(87.5)	1.289	0.687–2.418
Pots	11(78.6)	0.167	0.167–2.280
Gallons	33(86.8)	1.146	0.428–3.066
Sachet water users	10(52.6)	0.166	0.064–0.429*
Water treatment practices			
Boiling	89(79.5)	0.546	0.307–0.907*
Sedimentation	24(77.4)	0.557	0.228–1.359
Filtration	23(71.9)	0.398	0.174–0.909*
Chlorination	6(100)	0.851	0.817–1.887
Other methods	6(100)	0.851	0.817–1.887
Sanitation level			
Very good	31(59.6)	0.179	0.094–0.343*
Good	141(83.4)	0.769	0.442–1.339
Poor	162(94.7)	4.945	2.358–10.371*
Very poor	10(90.9)	1.737	0.218–13.823

Note: Odd ratios are relative to “No (i.e. negative response)” responses. * Significant at p < 0.05, CI = Confidence interval.

The people whose water choices were decided based on quality and reliability had a greater than 50% reduced chance of WBDs compared to those who made no such considerations (OR = 0.476; CI_95_: 0.246–0.921). Boiling and filtration were the two water treatment practices that significantly reduced the odds of WBDs in the population. Poor sanitation was the most important determinant of WBDs. Those whose sanitary practices were poor had approximately 400% greater chances of WBDs compared to those whose sanitary practices were not poor (OR = 4.945; CI_95_: 2.358–10.371). People whose hygiene level was very good had significantly reduced risk of WBDs. Further analysis on risk assessment and association of respondents' water choices, knowledge and practices to the different waterborne pathogens reported in the study population are highlighted on Table S3, S4 and S5.

## 4. Discussion

The overall prevalence of waterborne infection in the study population (85.4%) revealed that the microbiological quality of household drinking water in Enugu Urban, Nigeria was very poor, which is common in most parts of developing countries, in both urban and rural settings [13],[23]–[26]. Factors responsible for such poor household water quality and WBD prevalence include unimproved water supply, poor sanitation level, mothers' limited level of education, improper hygiene**,** lack of child health care by caregivers, low immunity, and unguided playing habits, especially among children below 5 years of age, in developing countries [14],[27]–[31]. Most communities in the study area use communal source for water collection that are exposed to contamination mainly by domestic and fecal waste from humans and, in a few cases, animals. The age disparity in prevalence was not significant, aligning this finding with our previous work on the retrospective distribution of waterborne disease in the same study location where we reported higher waterborne disease incidence in younger age groups, especially children below 5 years of age, when compared to the older age groups [16]. We could extrapolate from these two studies that older individuals are more likely to be asymptomatic carriers of WBD etiological agents, increasing the risk of susceptibility of the younger age groups. The high prevalence of *E. coli* in comparison with other pathogens is an indicator of high microbial contamination of these drinking water sources. The seasonal prevalence of the WBD aligns with our previous retrospective reports [16], underlining the dire need for public health intervention, especially during the dry seasons.

One of the major problems faced in most developing countries is the lack of a functional water project scheme; communities do not have regular and functional water projects. Most vulnerable households resort to questionable sources of water to satisfy their requirement for drinking water, thereby increasing the rate of waterborne diseases. This survey identified that more than half of the study population (53.6%) depends on water from private borehole dealers distributed mainly by tankers or vendors. Some households often use other water sources such as private well, public well, and municipal (a water project scheme managed by water corporation in the state; this source is often not available in all localities in most part of the country). Other sources are rainwater and sachet/bottle water which depend on the economic status of the household, since most water sources have prices which varies depending on the quality of the water, as well as the technology required to access the water. Risk perception from household drinking water choice greatly varies; some households choose their drinking water because of convenience and its availability, not necessarily based on the quality and risk associated with the water. As reported from this study, almost all the study population (90.6%) had to make their choice because of its availability and convenience. Households must select a water source from limited options. This choice is subject to budget constraints and availability [32]. Few of the respondents identified that their water choices were dependent on quality/reliability and average perception of water safety (15.6% and 26.8% respectively). This implies that most households do not consider risk associated with a water source when making their choice. This can be related to the fact that most of these water sources are inconsistent, making most of the households to choose any water reachable and affordable for them.

Assessment of the household water choices in association with prevalence of waterborne diseases in the study population indicates that respondents who source water from public wells and boreholes/vendors had greater risk of having a waterborne disease when compared with other choices. This finding can be directly related to the fact that these are unimproved water sources that are dependent on a water carriage system. Distance and personal hygiene of the carrier can increase vulnerability to contamination at many points in its journey from reservoir to mouth. Stream and sachet water users had lesser odds of having waterborne diseases than other water choices. This is questionable among stream water users but can be associated to the fact that some of the households that depended on the stream water are those living in the urban slum and may have depended entirely on the stream water for survival and thus, may have acquired immunity towards waterborne pathogens. Also, we extrapolate that the reduced risk among river/stream water drinkers compared to the non-drinkers was probably due to tendency to treat such water. Such tendency results from knowledge of contaminated nature of the water, and the fear of disease exposure. Most of the respondents that chose their water based on its quality and reliability were significantly less infected with waterborne diseases or pathogens than those who chose their water sources for other reasons. This agrees with the findings that most households deliberately choose water that they perceive to have less associated risk to waterborne diseases [33].

Access to safe water and sanitation facilities as well as knowledge of proper hygienic practices can reduce the risk of illness and death from waterborne diseases [34]. More than half of the study population (63.5%) had no knowledge of water treatment and did not practice any water treatment method. This implies that majority of the respondent generally perceived that their water source was good for drinking. This aligning with Onjala et al., [33] which explains that the riskier the individuals perceive the water source to be, the more likely they are to treat the water from that source. Water boiling was identified as the main method of water treatment among respondents. This method may be popular due to its ease and due to lack of knowledge of other approved water treatment methods [34]. Although water boiling is one of the easiest water treatment practices, it can be ineffective if not done properly.

Most of the respondents identified diarrhea (94.3%) as the major waterborne disease; their response may be attributed to its high prevalence, consequences, and its many associated etiological agents. Due to the inconsistent nature of drinking water in Enugu Urban, only 4.7% of the population practices no water storage method because they totally depended on sachet/bottle water. The type of vessel used to store drinking water affects its safety because wide-mouthed containers like buckets (with or without lid) and pots, which accounted for 56.2% of used vessels, are more prone to contamination because of their large open surface area. Not only were the buckets and pots left open, but users often dip cups and jugs in them to directly draw the water, enhancing the risks of fecal contamination. Furthermore, children can easily play with the exposed water. These findings are consistent with other similar research elsewhere that has shown that even drinking water that is safe at the source, is subject to frequent and extensive fecal contamination during collection, storage and use in the home [35]. Households that store their water with a plastic container without a lid and those that have poor hygiene level also had greater risk of waterborne diseases while respondents that did not store their drinking water, that always boil and filter their water had lesser risk of waterborne infection. This justifies the hypothesis that there are varieties of pathways for fecal matters to contaminate stored water in the home [15]. Earlier studies by Dunker [36] and Nala et al. [37] have shown that open water storage containers were more at risk of being contaminated by human and animals than containers which were covered.

## 5. Conclusion

This study shows that most household water choices had associated risks of waterborne infection, and emphasizes the need for proper “point of use” household water treatment and storage (HWTS) as highlighted by Lantagne et al. [38]. HWTS systems are proven, low-cost interventions that have the potential to provide safe water to those who may not have access to safe water sources in the near term, and thus significantly reduce morbidity due to waterborne diseases and improve the quality of life. The study strongly recommends the following:

1. Provision of safe, consistent, and easily accessible potable water sources that will improve the general welfare of Enugu Urban and reduce the risk of waterborne diseases.

2. The effectiveness of point of use household water treatment and storage should be prioritised by educating the population on the importance of a multi-barrier water treatment approach which uses combinations of technologies and the use of tested and improved water storage containers approved by Centre for Disease and Control [39].

3. Agencies that oversee public hygiene and health issues in the urban centres should provide and enforce management guidelines for household water choices and sanitation level.

4. Lastly, government should give closer attention to urban water supply, sanitation, and hygiene policy. Ensuring that appropriate measures are taken to improve access to safe drinking water, sanitation, and hygiene in the state. Since the availability of both potable water and adequate sanitation has potential to prevent at least 9.1% of the global disease burden and 6.3% of all deaths [40].
