# Supplementary material for: Risk assessment of waterborne infections in Enugu State, Nigeria: Implications of household water choices, knowledge, and practices
Source: AIMS Public Health. 2020 Aug 6;7(3):634–49. doi: 10.3934/publichealth.2020050 (PMC7505784; doi:10.3934/publichealth.2020050)
Supplement: Supplementary file 1 [file publichealth-07-03-050-s001.pdf]

AIMS Public Health
http://www.aimspress.com/journal/aimsph

AIMS Public Health, 7(3): 634–649.
DOI: 10.3934/publichealth.2020050
Received: 05 July 2020
Accepted: 27 July 2020
Published: 06 August 2020

Research article

Risk assessment of waterborne infections in Enugu State, Nigeria: Implications of household water choices, knowledge, and practices

Onyekachi Juliet Okpasuo[1,]*, Ifeanyi Oscar Aguzie[1], Anunobi Tochukwu Joy[2] and Fabian C Okafor[1,3]

[1] Parasitology and Public Health Research Laboratory, University of Nigeria, Nsukka, Nigeria
[2] Science Laboratory Technology Department, Federal Polytechnic, Idah, Kogi State, Nigeria
[3] Ecology and Environmental Biology, University of Nigeria, Nsukka, Nigeria

* **Correspondence:** Email: onyekachi.okpasuo@unn.edu.ng.

Supplementary

Table S1. Co-infections prevalence of waterborne etiological agents in Enugu Urban, Nigeria (number examined = 403).

Coinfections	Number infected	Prevalence (%)
Giardia/E. Histolytica	27	6.7
Giardia/Cryptosporidium	5	1.2
Giardia/Salmonella	9	2.2
Giardia/Shigella	6	1.5
Giardia/E.coli	22	5.5
Giardia/Proteus	4	1.0
Giardia/Enterobacter	4	1.0
Giardia/Klebisella	4	1.0
E. histolytica/Cryptosporidium	2	0.5
E. histolytica/Salmonella	7	1.7
E. histolytica/Shigella	2	0.5

Continued on next page

Coinfections	Number infected	Prevalence (%)
E. histolytica/E.coli	26	6.5
E. histolytica/Proteus	5	1.2
E. histolytica/Enterobacter	2	0.5
E. histolytica/Klebisella	5	1.2
Cryptosporidium/Salmonella	3	0.7
Cryptosporidium/Shigella	1	0.2
Cryptosporidium/E.coli	2	0.5
Cryptosporidium/Kebisella	1	0.2
Salmonella/Shigella	4	1.0
Salmonella/E.coli	18	4.5
Salmonella/Proteus	11	2.7
Salmonella/Enterobacter	1	0.2
Salmonella/Klebisella	4	1.0
Shigella/E.coli	2	0.5
Shigella/Proteus	1	0.2
Shigella/Enterobacter	1	0.2
Shigella/Klebisella	3	0.7
E.coli/Proteus	8	2.0
E.coli/Enterobacter	4	1.0
E.coli/Klebisella	5	1.2
Proteus/Enterobacter	2	0.5
Proteus/Klebisella	2	0.5
Enterobacter/Klebisella	1	0.2
E. histolytica/E. coli/Proteus	3	0.7
Giardia/E. histolytica/E.coli	3	0.7
Giardia/E.histolytica/Salmonella	6	1.5
E. histolytica/E.coli/Salmonella	5	1.2
Giardia/Salmonella/Shigella	17	4.2
E. histolytica/Salmonella/Proteus	2	0.5
Giardia/Salmonella/E.coli	2	0.5
Giardia/E. histolytica/Klebisella	2	0.5
Giardia/E. coli/Proteus	4	1.0
Giardia/E. histolytica/Shigella	2	0.5
Giardia/E. histolytica/Proteus	1	0.2

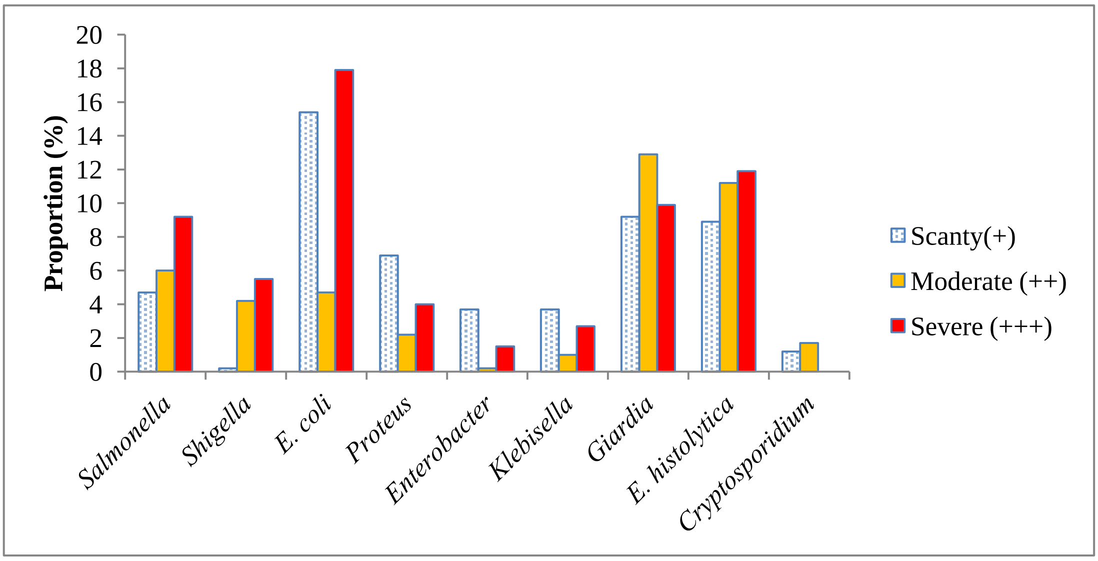

Figure S1. Intensity of isolated waterborne disease aetiological agents from Enugu Urban, Nigeria.

Table S2. Risk assessment and association of respondent's water choices, knowledge and practices to the different waterborne pathogens (*Giardia* spp., *E. histolytica* and *Cryptosporidium* spp.,) in the study population (Number examined = 403).

Characteristics	*Giardia* spp.			*E. histolytica*			*Cryptosporidium* spp.		
**Water choices**	**NI (%)**	**OR**	**95% CI**	**NI (%)**	**OR**	**95% CI**	**NI (%)**	**OR**	**95% CI**
Drinking water choices									
Municipal	33(32.7)	0.859	0.533,1.385	39(36.5)	1.241	0.774,1.988	6(5.9)	1.208	0.458,3.203
Private well	3(18.8)	0.412	0.115,1.470	2(12.5)	0.279	0.063,1.247	1(6.2)	1.223	0.157,9.730
Public well	63(40.6)	1.465	0.965,2.223	48(31)	0.860	0.560,1.320	10(8.5)	1.486	0.616,3.586
Borehole /vendors	78(36.1)	1.086	0.721,1.637	81(37.5)	1.558	1.021,2.376*	6(2.8)	0.328	0.124,0.862*
Stream/rivers	6(18.8)	0.399	0.160,0.993*	7(21.9)	0.544	0.229,1.293	2(6.2)	1.235	0.274,5.557
Rainwater	27(36.5)	1.069	0.633,1.807	23(31.1)	0.898	0.522,1.545	5(6.8)	1.418	0.500,4.000
Sachet water	29(33.3)	0.898	0.544,1.483	27(31)	0.892	0.535,1.486	3(3.4)	0.591	0.170,2.056
Reason for the choice									
Price	24(32.4)	0.858	0.502,1.467	17(23)	0.548	0.304,0.988*	9(12.2)	3.658	1.480,9.037*
Distance	2(40)	1.229	0.203,7.440	3(60)	3.092	0.510,18.734	0(0)	0.947	0.929,6.969
Quality and reliable	18(28.6)	0.697	0.386,1.256	16(25.4)	0.649	0.353,1.194	1(1.6)	0.258	0.34,1.958
Available	133(36.4)	1.847	0.849,4.020	122(33.4)	1.232	0.591,2.507	19(5.2)	0.988	0.221,4.416
Average perception of water safety	34(31.5)	0.796	0.497,1.273	30(27.8)	0.717	0.442,1.164	3(2.8)	0.44	0.127,1.524
**Knowledge**	**NI (%)**	**OR**	**95% CI**	**NI (%)**	**OR**	**95% CI**	**NI (%)**	**OR**	**95% CI**
Knowledge of waterborne diseases									
Diarrhoea	136(35.8)	1.579	0.608,4.100	127(33.4)	1.422	0.547,3.695	19(5.0)	0.553	0.553,1.121
Dysentery	45(40.9)	1.399	0.891,2.197	40(36.4)	1.229	0.776,1.229	6(5.5)	1.069	0.404,2.830
Cholera	46(39.6)	1.288	0.807,1.961	38(32.2)	0.950	0.601,1.502	3(2.5)	0.387	0.112,1..339
Typhoid	80(39.8)	1.483	0.990,2.252	61(30.3)	0.787	0.519,1.193	8(4)	0.603	0.244,1,487
Others	13(27.7)	0.673	0.343,1.321	14(29.8)	0.845	0.435,1.639	1(2.1)	0.365	0.048,2,786
Don't know	5(33.3)	0.916	0.307,2.734	4(26.7)	0.730	0.228,2.338	3(20)	5.139	1.331,19.837*

Continued on next page

Characteristics	*Giardia* spp.			*E. histolytica*			*Cryptosporidium* spp.		
**Knowledge**	**NI (%)**	**OR**	**95% CI**	**NI (%)**	**OR**	**95% CI**	**NI (%)**	**OR**	**95% CI**
Knowledge of factors causing the disease									
Dirty environments	87(32.3)	0.687	0.447,1.054	88(32.7)	0.962	0.962,1.493	13(4.8)	0.800	0.323,1.979
Unhygienic practices	76(34.4)	0.921	0.611,1.389	69(31.2)	0.837	0.552,1.269	13(5.9)	1.359	0.551,3.355
Drinking contaminated water	137(35.3)	1.092	0.366,3.258	129(33.2)	1.370	0.428,4.385	18(4.6)	0.195	0.050,0.751*
No idea	5(33.3)	0.916	0.307,2.734	4(26.7)	0.730	0.228,2.338	3(20.0)	5.139	1.331,19.837
Knowledge of water treatment									
Have Knowledge of water treatment methods	42(28.6)	0.624	0.403,0.966*	36(24.5)	0.532	0.338,0.836*	12(8.2)	2.445	1.002,5.937*
**Practices**	**NI (%)**	**OR**	**95% CI**	**NI (%)**	**OR**	**95% CI**	**NI (%)**	**OR**	**95% CI**
Water storage practices									
Plastic bucket with lid	51(38.1)	1.202	0.781,1.849	54(40.3)	1.623	1.052,2.505*	5(3.7)	0.613	0.220,1.710
Plastic bucket without lid	34(43.6)	1.553	0.938,2.569	26(33.3)	1.019	0.603,1.721	8(10.3)	2.743	1.095,6.869
Tanks	45(37.5)	1.151	0.738,1.793	35(29.2)	0.777	0.489,1.236	6(5)	0.940	0.356,2.485
Pots	7(50)	1.881	0.647,5.476	6(42.9)	1.547	0.526,4.554	0(0)	0.946	0.924,0.969
Gallons	3(7.9)	0.139	0.042,0.462*	8(21.1)	0.512	0.228,1.150	1(2.6)	0.466	0.061,3.574
None(sachet water users)	2(10.5)	0.205	0.047,0.901*	4(21.1)	0.527	0.171,1.621	1(5.3)	1.011	0.128,7.960
Water treatment practices									
Boiling	35(31.2)	0.782	0.491,1.245	31(27.7)	0.709	0.439,1.145	9(8.0)	2.032	0.832,4.964
Sedimentation	9(29.0)	0.735	0.329,1.643	7(22.6)	0.569	0.239,1.358	3(9.7)	2.107	7.589,0.585
Filtration	11(34.4)	0.960	0.449,2.052	5(15.6)	0.352	0.132,0.935*	2(6.2)	1.235	0.274,5.557
Chlorination	5(83.3)	9.489	1.092,82.033*	4(66.7)	4.155	0.751,22.980	0(0)	0.947	0.925,0.969
Other methods	5(83.1)	9.489	1.092,82.033*	4(66.7)	4.155	0.751,22.980	0(0)	0.947	0.925,0.969
Sanitation level									
Very good	9(17.3)	0.343	0.162,0.726*	12(23.1)	0.570	0.288,1.127	2(3.8)	0.699	0.188,3.092
Good	56(33.1)	0.853	0.562,1.293	56(33.1)	1.010	0.663,1.539	9(5.3)	1.041	0.428,2.529
Poor	77(45.0)	2.105	1.389,3.190*	63(36.8)	1.350	0.888,2.052	10(5.8)	1.248	0.518,3.009
Very poor	0(0)	0.638	0.687,0.592	2(18.2)	0.443	0.094,2.079	0(0)	0.946	0.924,0.969

Note: NI = Number infected, OR = Odd ratio, CI = Confidence interval, * Significant at $p < 0.05$.

Table S3. Risk assessment and association of respondent's water choices, knowledge and practices to the different waterborne pathogens (*Shigella* spp., *E. coli* and *Proteus* spp.) in the study population (Number examined = 403).

Characteristics	*Shigella* spp.			*E. coli*			*Proteus* spp.		
**Water choices**	**NI (%)**	**OR**	**95% CI**	**NI (%)**	**OR**	**95% CI**	**NI (%)**	**OR**	**95% CI**
Drinking water choices									
Municipal	4(25)	3.352	1.026,10.950*	7(43.8)	1.284	0.468,3.521	0(0)	0.863	0.829,0.896
Public well	11(7.1)	0.600	0.290,1.243	71(45.8)	1.711	1.33,2.584*	24(15.5)	1.384	0.773,2.477
Private well	4(25)	3.352	1.026,10.950	7(43.8)	1.284	1.133,0.468	0(0)	0.863	0.829,0.898
Borehole /vendors	24(11.1)	1.433	0.728,2.821	85(39.4)	1.135	0.758,1.701	27(12.5)	0.885	0.496,1.577
Stream/rivers	3(9.4)	0.963	0.279,3.318	11(34.4)	0.845	0.395,1.804	3(9.4)	0.664	0.195,2.262
Rainwater	1(1.4)	0.105	0.104,0.777*	35(47.3)	1.605	0.965,2.670	10(13.5)	1.039	0.496,2.177
Sachet water	12(13.8)	1.713	0.829,3.539	22(25.3)	0.478	0.281,0.841*	14(16.1)	1.362	0.702,2.643
Reason for the choice									
Price	3(4.1)	0.344	0.103,1.149	31(41.9)	1.223	0.180,6.601	4(5.4)	0.327	0.114,0.935*
Distance	1(20)	2.368	0.258,21.734	2(40.0)	1.091	0.305,1.007	0(0)	0.867	0.834,0.901
Quality and reliable	11(17.5)	2.357	1.106,5.024*	17(27)	0.554	0.667,2.790	10(15.9)	1.303	0.617,2.752
Available	33(9.0)	0.530	0.207,1.360	141(38.6)	1.364	0.568,1.418	48(13.2)	0.999	0.372,2.685
Average perception of water safety	18(16.7)	2.610	1.331,5.115*	39(36.1)	0.897	0.456,1.067	15(13.9)	1.091	0.573,2.075
**Knowledge**	**NI (%)**	**OR**	**95% CI**	**NI (%)**	**OR**	**95% CI**	**NI (%)**	**OR**	**95% CI**
Knowledge of waterborne diseases									
Diarrhoea	37(9.7)	1.133	0.255,5.023	139(36.6)	0.371	0.156,0.879*	50(13.2)	1.010	0.290,3.524
Dysentery	9(8.2)	0.781	0.358,1.703	42(38.2)	1.013	0.645,1.590	9(8.2)	0.504	0.237,1.071
Cholera	12(10.2)	1.082	0.528,2.215	46(39)	1.063	0.684,1.652	14(11.9)	0.849	0.442,1.630
Typhoid	20(10)	1.064	2.060,0.550	75(37.3)	0.946	0.633,1.415	24(11.9)	0.809	0.453,1.445
Others	7(14.9)	1.772	0.734,4.277	22(46.8)	1.511	0.820,2.788	6(12.8)	0.962	0.387,2.390
Don't know	1(6.7)	0.658	0.084,5.142	7(46.7)	1.450	0.515,4.083	1(6.7)	0.462	0.590,3.584

Continued on next page

Characteristics	*Shigella* spp.			*E. coli*			*Proteus* spp.		
**Knowledge**	**NI (%)**	**OR**	**95% CI**	**NI (%)**	**OR**	**95% CI**	**NI (%)**	**OR**	**95% CI**
Knowledge of factors causing the disease									
Dirty environments	31(11.5)	2.051	0.919,4,596	97(36.1)	0.786	0.514,1.200	40(14.9)	1.626	0.837,3.156
Unhygienic practices	28(12.7)	2.255	1.090,4.667*	81(36.7)	0.884	0.590,1.324	32(14.5)	1.298	0.720,2.340
Drinking contaminated water	38(9.8)	1.520	0.194,11.881	146(37.6)	0.689	0.245,1.941	52(13.4)	2.167	0.279,16.825
No idea	1(6.7)	0.658	0.084,5.142	7(46.7)	1.450	0.515,4.083	1(6.7)	0.462	0.059,3.584
Knowledge of water treatment									
Have Knowledge of water treatment methods	10(6.8)	0.571	0.270,1.209	48(32.7)	0.697	0.456,1.067	17(11.6)	0.799	0.432,1.480
**Practices**	**NI (%)**	**OR**	**95% CI**	**NI (%)**	**OR**	**95% CI**	**NI (%)**	**OR**	**95% CI**
Water storage practices									
Plastic bucket with lid	7(5.2)	0.408	0.175,0.915*	51(38.1)	1.006	0.656,1.542	15(11.2)	0.766	0.405,1.449
Plastic bucket without lid	8(10.3)	1.084	0.497,2.462	30(38.5)	1.026	0.617,1.706	13(16.7)	1.425	0.721,2.816
Tanks	17(14.2)	1.958	0.999,3.837*	48(40)	1.130	0.730,1.751	12(10)	0.656	0.332,1.297
Pots	0(0)	0.900	0.870,0.930	6(42.9)	1.235	0.420,3.629	2(14.3)	1.105	0.240,5.079
Gallons	4(10.5)	1.093	0.392,3.309	16(42.1)	1.210	0.614,2.384	9(23.7)	2.264	1.006,5.097*
None (sachet water users)	3(15.8)	1.813	0.509,6.519	2(10.5)	0.182	0.041,0.797*	2(10.5)	0.768	0.172,3.424
Water treatment practices									
Boiling	6(5.4)	0.443	0.180,1.087	39(34.8)	0.829	0.527,1.307	14(12.5)	0.923	0.480,1.775
Sedimentation	3(9.7)	1.00	0.290,3.453	8(25.8)	0.545	0.237,1.250	2(6.5)	0.434	0.101,1.875
Filtration	1(3.1)	0.283	0.380,2.130	13(40.6)	1.129	0.54,2.357	2(6.2)	0.418	0.097,1.804
Chlorination	0(0)	0.902	0.873,0.932	3(50)	1.647	0.328,8.264	0(0)	0.866	0.834,0.901
Other methods	0(0)	0.902	0.873,0.932	3(50)	1.647	0.328,8.264	0(0)	0.866	0.834,0.901
Hygiene level									
Very good	6(11.5)	1.257	0.499,3.164	11(21.2)	0.395	0.196,0.794*	3(5.8)	0.369	0.111,1.228
Good	14(8.3)	0.755	0.380,1.500	61(36.1)	0.872	0.579,1.312	17(10.1)	0.615	0.333,1.137
Poor	18(10.5)	1.182	0.609,2.294	76(44.4)	1.610	1.072,2.419*	31(18.1)	2.114	1.175,3.801*
Very poor	1(9.1)	0.932	0.116,7.477	5(45.5)	1.374	0.472,4.581	2(18.2)	1.486	0.312,1.137

Note: NI = Number infected, OR = Odd ratio, CI = Confidence interval, * Significant at $p < 0.05$

Table S4. Risk assessment and association of respondent's water choices, knowledge and practices to the different waterborne pathogens (*Klebisella* spp., *Enterobacter* spp. and *Salmonella* spp.) in the study population.

Characteristics	*Klebisella* spp.			*Enterobacter* spp.			*Salmonella* spp.		
**Water choices**	**NI (%)**	**OR**	**95% CI**	**NI (%)**	**OR**	**95% CI**	**NI (%)**	**OR**	**95% CI**
Drinking water choices									
Municipal	1(6.2)	0.823	0.105,6.453	2(12.5)	2.621	0.557,12.332	3(18.8)	0.929	0,560,1.752
Private well	13(8.4)	1.244	0.587,2.638	6(3.9)	0.584	0.223.,1.526	3(18.8)	0.929	0.574,1.575
Public well	1(6.2)	0.823	0.105,6.453	2(12.5)	2.261	0.557,12.332	30(19.4)	0.950	0.258,3.34
Borehole /vendors	17(7.9)	1.143	0.540,2.421	10(4.6	0.708	0.299,1.678	44(20.4)	1.073	0.258,3.341
Stream/rivers	4(12.5)	1.896	0.618,5.815	2(6.2)	1.170	0.261,5.347	7(21.9)	1.143	0.656,1.754
Rainwater	8(10.8)	1.691	0.722,3.964	4(5.4)	0.987	0.324,3.008	18(24.3)	1.384	0.496,2.746
Sachet water	3(3.4)	0.382	0.113,1.291	5(5.7)	1.072	0.384,2.994	14(16.1)	0.726	0.386,1.386
Reason for the choice									
Price	6(8.1)	1.121	0.441,2.849	5(6.8)	1.330	0.474,3.728	18(24.3)	1.384	0.761,2.519
Distance	0(0)	0.925	0.899,0.951	0(0)	0.945	0.923,0.967	0(0)	0.799	0.761,0.839
Quality and reliable	2(3.2)	0.365	0.085,1.574	5(7.9)	1.638	0.582,4.614	15(23.8)	1.322	0.697,2.507
Available	28(7.7)	1.496	0.342,6.538	20(5.5)	1.043	0.234,4.646	70(19.2)	0.664	0.508,1.432
Average perception of water safety	6(5.6)	0.664	0.264,1.672	6(5.6)	1.026	0.391,2.693	25(23.1)	1.314	0.770,2.243
Knowledge	NI (%)	OR	95% CI	NI (%)	OR	95% CI	NI (%)	OR	95% CI
Knowledge of waterborne diseases									
Diarrhoea	27(7.1)	0.510	0.142,1.825	22(5.8)	1.061	1.035,1.088	76(20)	1.188	0.392,3.593
Dysentery	6(5.5)	0.647	0.257,1.627	5(4.5)	0.773	0,298,2.149	20(18.2)	0.863	0.492,1.513
Cholera	6(5.1)	1.583	0.232,1.464	8(6.8)	1.408	0.574,3.450	19(16.1)	0.705	0.400,1.242
Typhoid	12(6.0)	0.649	0.304,1.385	13(6.5)	1.483	0.619,3,551	36(17.9)	0.783	0.479,1.281
Others	3(6.4)	0.831	0.242,2.853	4(8.5)	1.747	0.565,5.402	7(14.9)	0.678	0.292,1.577
Don't know	2(13.3)	1.976	0.425,9.204	0(0)	0.943	0.921,0.967	2(13.3)	0.611	0.135,2.766

Continued on next page

Characteristics	*Klebisella* spp.			*Enterobacter* spp.			*Salmonella* spp.		
**Knowledge**	**NI (%)**	**OR**	**95% CI**	**NI (%)**	**OR**	**95% CI**	**NI (%)**	**OR**	**95% CI**
Knowledge of factors causing the disease									
Dirty environments	21(7.8)	1.176	0.523,2.644	15(5.6)	1.071	0.426,2.694	53(19.7)	0.972	0.579,1.633
Unhygienic practices	13(59)	0.607	0.286,1.285	12(5.4)	0.988	0.417,2.341	42(19.0)	0.889	0.544,1.452
Drinking contaminated water	28(7.2)	0.506	0.109,2.352	22(5.7)	1.060	1.035,1.086	78(20.1)	1.635	0.362,7.398
No idea	2(13.3)	1.978	0.425,9.204	0(0)	0.921	0.921,0.967	2(13.3)	0.611	0.135,2,766
Knowledge of water treatment									
Have Knowledge of water treatment methods	11(7.5)	1.009	0.466,2.183	7(4.8)	0.803	0.320,2.018	31(21.1)	1.129	0.682,1.869
**Practices**	**NI (%)**	**OR**	**95% CI**	**NI (%)**	**OR**	**95% CI**	**NI (%)**	**OR**	**95% CI**
Water storage practices									
Plastic bucket with lid	10(7.5)	1.004	0.456,2.210	6(4.5)	0.741	0.283,1.940	27(20.1)	1.028	0.613,1.727
Plastic bucket without lid	6(7.7)	1.045	0.412,2.615	5(6.4)	1.241	0.443,3.473	20(25.6)	1.523	0.852,2.721
Tanks	8(6.7)	0.847	0.366,1.961	6(5.0)	0.878	0.335,2.302	21(17.5)	0.805	0.464,1.398
Pots	0(0)	0.923	0.897,0.950	1(7.1)	1.348	0.168,10.800	3(21.4)	1.105	0.301,4.058
Gallons	6(15.8)	2.664	1.015,6.994*	3(7.9)	1.561	0.440,5.538	6(15.8)	0.737	0.297,1.829
None (sachet water users)	0(0)	0.922	0.895,0.949	1(5.3)	0.960	0.122,7.543	3(15.8)	1.813	0.504,6.519
Water treatment practices									
Boiling	8(7.1)	0.941	0.406,2.179	5(4.5)	0.753	0.271,2.093	24(21.4)	1.144	0.669,1.959
Sedimentation	3(9.7)	1.369	0.391,4.795	0(0)	0.941	0.917,0.965	8(25.8)	1.449	0.623,3.373
Filtration	1(3.1)	0.380	0.082,2.888	2(6.2)	1.170	0.261,5.247	4(12.5)	0.55	0.189,1.629
Chlorination	0(0)	0.924	0.899,0.951	0(0)	0.945	0.922,0.967	0(0)	0.798	0.760,0.839
Other methods	0(0)	0.924	0.899,0.951	0(0)	0.945	0.922,0.967	0(0)	0.798	0.760,0.839
Hygiene level									
Very good	1(1.9)	0.218	0.029,1.633	4(7.7)	1.542	0.501,4.748	7(13.5)	0.592	0.257,1.368
Good	11(6.5)	0.788	0.365,1.702	11(6.5)	1.411	0.597,3.336	42(24.9)	1.706	1.043,2.791*
Poor	15(8.8)	1.391	0.661,2.929	7(4.1)	0.617	0.246,1.549	28(16.4)	0.678	0.407,1.128
Very poor	3(27.3)	5.069	1.271,20.217*	0(0)	0.944	0.921,0.967	3(27.3)	1.534	0.398,5.918

Note: NI = Number infected, OR = Odd ratio, CI = Confidence interval, * Significant at $p < 0.05$.

AIMS Press

© 2020 the Author(s), licensee AIMS Press. This is an open access article distributed under the terms of the Creative Commons Attribution License (http://creativecommons.org/licenses/by/4.0)
